# Paraptosis, a new type of cell death that can be applied to cancer treatment

**DOI:** 10.3389/fonc.2026.1959542

**Published:** 2026-10-05

**Authors:** Xudong Wang, Jiaxing Ma, Bowen Tan, Yang Wang, Zhengwan Wang, Chunlan Hou, Qingjuan Chen, Xiang Jin

**Affiliations:** 1 Department of Orthopedics, Junlian County People’s Hospital, Yibin, Sichuan, China; 2 West China School of Medicine, Sichuan University, Chengdu, China; 3 Department of Breast Surgery, North Sichuan Medical College, Nanchong, Sichuan, China; 4 Department of Oncology, 3201 Hospital, Hanzhong, China; 5 Department of Breast Surgery, Yibin First People’s Hospital, Yibin, Sichuan, China; 6 Department of Breast Surgery, Junlian County People’s Hospital, Yibin, Sichuan, China

**Keywords:** cancer therapy, cell death, endoplasmic reticulum, molecular mechanisms, paraptosis

## Abstract

Paraptosis is a form of non-apoptotic cell death characterized by cytoplasmic vacuolization and swelling of the mitochondria and endoplasmic reticulum (ER). Recent studies have demonstrated that inducing paraptosis in cancer cells can circumvent apoptosis resistance and synergize with other forms of cell death and tumor immunity, providing a novel strategy for anti-tumor therapy. The underlying mechanism of paraptosis involves ER stress triggered by inducers, leading to the swelling of the ER and mitochondria. This subsequently alters the cytoskeleton and disrupts molecular processes, such as calcium (Ca^2+^) homeostasis, ultimately causing cytoplasmic vacuolization and cell death. Unlike other forms of programmed cell death, paraptosis is caspase-independent and is marked by mitochondrial and ER swelling, along with extensive cytoplasmic vacuolization, without nuclear pyknosis or cell membrane rupture. Various natural compounds, chemical agents, and nanomedicines have been shown to induce paraptosis, which also holds potential in immunotherapy by promoting immunogenic cell death (ICD) and systemic anti-tumor responses. Thus, in cases where cancer cells evade and develop resistance to conventional pro-apoptotic therapies, paraptosis induction offers an alternative therapeutic approach. A comprehensive understanding of the molecular mechanisms underlying paraptosis and its targeted induction may facilitate the development of novel cancer treatments. This paper explores the impact of paraptosis on cancer cells, the mechanisms by which different drugs induce paraptosis, and the potential applications of paraptosis in cancer therapy.

## 1 Introduction

Paraptosis is a cell death pathway characterized by vacuolization and damage to the endoplasmic reticulum (ER) and mitochondria (1). Since there is currently no definitive and specific marker or induction mechanism for paraptosis, it has not yet been recognized as a distinct form of programmed cell death by the Nomenclature Committee on Cell Death (2, 3). Nevertheless, its unique features, such as ER swelling and cytoplasmic vacuolization, differentiate it from other forms of programmed cell death, offering a novel approach for inducing cell death and a potential strategy for tumor treatment (4). Paraptosis exhibits morphological differences compared to apoptosis and autophagy, including cytoplasmic vacuolization originating from the ER, caspase independence, and the absence of apoptotic features (5). Paraptosis has been shown to be induced through various mechanisms, including the expression of insulin-like growth factor 1 receptor (IGF1R) (1), production of reactive oxygen species (ROS) (6), and Ca²^+^ influx into mitochondria or ion channel activation (7). These factors lead to extensive cytoplasmic vacuolation, including swelling of the ER and mitochondria (2), followed by molecular changes that ultimately result in cancer cell death.

Current methods of utilizing cell death for tumor treatment are limited and often one-dimensional. A significant challenge is the ability of tumor cells to escape apoptosis, which is considered a major cause of drug resistance and treatment failure. Paraptosis offers a novel approach to inducing cell death, providing a potential new strategy for cancer treatment (4). Although cancer cells possess various anti-apoptotic mechanisms, the fundamental process of paraptosis differs from that of apoptosis (8). Therefore, when cancer cells exhibit the ability to evade apoptosis, inducing paraptosis may serve as an alternative mode of cell death (9). Furthermore, numerous studies have shown that sustained activation of paraptosis enhances tumor immunogenicity, leading to cell death and more effective elimination of cancer cells (10).

The study of paraptosis provides new insights into the cell death process and its role in development and degradation. It also contributes to the development of novel strategies for efficient cancer prevention and the treatment of malignant cancers. This paper primarily reviews the basic mechanisms of paraptosis, the compounds that induce it, and their therapeutic effects on various cancers.

## 2 History of the development of paraptosis

The history of research into paraptosis is not particularly long, given that this unique cell death modality was first formally characterized and named in the early 21st century (11, 12). To provide a clear overview of its developmental trajectory in the field of cancer biology and cell physiology, a brief chronology of landmark studies on paraptosis is illustrated in **Figure 1**. The term “paraptosis” was first introduced in 2000 by Sperandio et al., who observed that, unlike apoptosis, paraptosis does not involve loss of membrane integrity (1, 8). Starting in 2009, several researchers, including Feng-Jiao Zhang (13), Mi Jin Yoon (14), En-Bo Wang (15), and Kenta Yokoi (16), demonstrated that paraptosis can be induced by various pathways and substances, such as the activation of mitogen-activated protein kinases (MAPKs), c-Jun N-terminal kinases (JNKs), mitogen-activated protein kinase kinase 2 (MEK2), and p38. In 2012 and beyond, Mi Jin Yoon (7), Silvia Yumnam (17), In Young Kim (18), and others identified the depletion of ER Ca^2+^ and subsequent mitochondrial Ca^2+^ overload as crucial factors in the induction of paraptosis. These findings have provided new molecular insights into the mechanisms of cell death and proposed a novel therapeutic strategy for inducing cell death in malignant breast cancer cells resistant to proteasome inhibitors and/or traditional pro-apoptotic treatments. In both 2014 and 2018, Mi Jin Yoon et al. (14) and Haote Ha (19) respectively reported that AIP-1/Alix may play a negative role in some instances of paraptosis. Furthermore, Dongjoo Lee et al. (20) in 2016 and Fabrizio Fontana (21) in 2020 demonstrated that various natural compounds can induce paraptosis in tumor cells both *in vitro* and *in vivo*. These studies suggest that further research into novel natural products and their derivatives is needed to identify more potent apoptosis-inducing factors and to elucidate the key signaling pathways that regulate this form of cell death (**Figure 1**).

**Figure 1:**
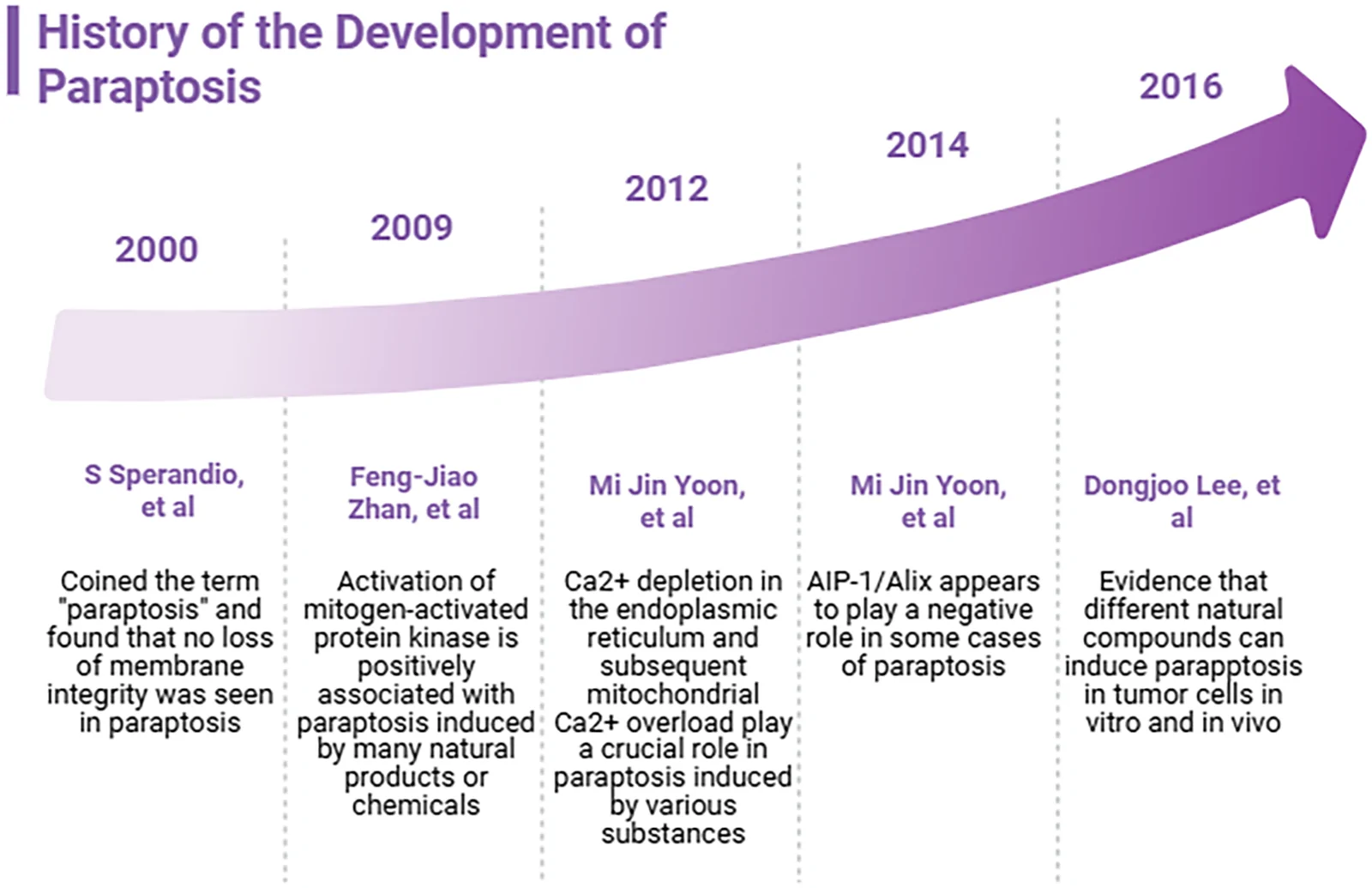
History of the development of paraptosis (2000–2016).

## 3 Characteristics of paraptosis

The primary feature of paraptosis is the dilated vacuolization of the ER and mitochondria, which occurs during development and in certain neurodegenerative conditions. Paraptosis is unresponsive to caspases inhibitors and Bcl-x(L) and is driven by Apaf-1-independent Caspase-9 activity. In the process of paraptosis, caspase-9 exerts non-enzymatic functions, which interacts with other proteins, for example, IGF-1R, to activate the MAPK signaling pathway (1, 12, 22). However, the process is still inhibited by caspases, which demonstrates that paraptosis does not rely on the proteolytic activity of caspase-9 (1, 3). Paraptosis differs significantly from several other well-recognized forms of cell death (**Table 1**), including apoptosis, autophagy, pyroptosis, iron death, copper death and so on, in terms of morphological features, initiating signals, regulatory molecular pathways and effector mechanisms. Unlike apoptosis, paraptosis does not exhibit typical signs such as nuclear fragmentation, apoptotic body formation, or chromatin condensation (9, 23). Its morphological features are more akin to necrosis, including mitochondrial swelling, aggregation, and an increased number and size of cytoplasmic vacuoles (10). In contrast, apoptotic cells undergo several characteristic biochemical changes, including protein cleavage, protein cross-linking, DNA fragmentation, and phagocytic recognition (24). Extensive protein cross-linking, achieved through tissue transglutaminase expression and activation, is another hallmark of apoptosis (25). Caspases play a central role in apoptosis, and cysteine asparaginases, which possess proteolytic activity, cleave proteins at aspartic acid residues (26, 27). In the mitochondrial pathway of apoptosis, intrinsic stress activates the pro-apoptotic proteins of the Bcl-2 family, which regulate mitochondrial outer membrane permeabilization (MOMP), and then induce the release of cytochrome c. Cytochrome c forms a complex with Apaf-1, constituting the apoptosome, which recruits and activates caspase-9 (28). Different caspases vary in their specificity for recognizing adjacent amino acids. Once caspases are activated, they appear to irreversibly drive the cell towards death (29). In contrast, despite the mPTP-induced changes in mitochondrial membrane potential during paraptosis, outer mitochondrial membrane (OMM) remains intact, and thus cytochrome c fails to be released into the cytosol (30). Consequently, caspases cannot be activated, but caspase-9 can still exert non-enzymatic effects (1, 31).

**Table 1:** Differences between several modes of cell death.

Differences	Paraptosis	Apoptosis	Autophagy	Pyroptosis	Iron death	Copper death
Nuclear	No DNA fragmentation	DNA degradation	Degraded after being swallowed into autophagosomes	–	–	Chromatin rupture
Cell membrane	–	Membrane shrinkage invagination	–	Rupture of the cell membrane	Increased cell membrane density	Rupture of the cell membrane
Cytoplasm	Extensive cytoplasmic vacuolization occurred	Cytoplasm dehydration concentration	–	Form a large number of pyroptosis bodies, and cytoplasmic outflows	–	Granular deformation and cavitation deformation
Organelles	Mitochondrial or/and ER swelling	ER expanded and fused with cell membrane to form a bud-like protrusion on the surface of the membrane	Degraded after being swallowed into autophagosomes	Flow out with cytoplasm	Mitochondria became smaller, membrane density increased, and cristae decreased	Mitochondrial shrinkage, ER damage
Triggering signals	ROS overproduction; ER stress; Ca^2+^ overloading	DNA damage; ER stress; ROS overproduction; growth factor/nutrient deprivation; oncogenic stress; kinase inhibition; death receptor ligation	Nutrient and energy deprivation; Protein, organelle damage and ROS imbalance; cellular stress; Hypoxia	PAMPs/DAMPs; LPS; TNF; Granzymes; Mutations	Iron-dependent membrane lipid peroxidation; PUFA-containing phospholipid peroxidation;ROS/iron chemistry and enzymatic lipid peroxidation; GPX4 inactivation; Cysteine deprivation	Excess intracellular copper; Copper ionophores; Cu(II) reduced to Cu(I); Lipoylated protein aggregation; Fe-S cluster loss and proteotoxic stress
Key regulatory molecules	MAPK; CHOP; LC3B;Alix; ATP; GSH	Bcl-2 family proteins; Caspases; p53; Cytochrome c	ULK complex; VPS34 complex; ATG conjugation machinery; Selective autophagy receptors;	GSDMD; Caspases; Granzymes; NINJ1; ESCRT	GPX4; FSP1; ACSL4; SLC7A11;	FDX1; LIAS; DLAT; SLC31A1, ATP7A, ATP7B
Mechanism of action and biochemical changes	Independent of caspase, mitochondrial membrane permeability changes, ATP depletion, Ca^2+^ imbalance, K+ channel opening and so on (1)	After receiving the apoptotic signal, the interaction between the apoptotic regulatory molecules leads to the activation of proteolytic enzymes and then into the continuous reaction process (17)	Forms a double-layer membrane structure and encapsulates the components in the cytoplasm to form autophagosomes after receiving the autophagy induction signal (18)	It depends on inflammatory caspase and is accompanied by the release of many proinflammatory cytokines factors (19)	Catalyzes the lipid peroxidation of highly expressed unsaturated fatty acids on the cell membrane under the action of divalent iron or lipoxygenase, thereby inducing cell death (20)	Copper induces cell death by targeting acyl-TCA cycle proteins (21)

DNA, deoxyribonucleic acid; ER, endoplasmic reticulum; ATP, adenosine triphosphate; TCA, tricarboxylic acid; ROS, reactive oxygen species; MAPK, mitogen-activated protein kinase; CHOP, C/EBP homologous protein; LC3B, microtubule-associated protein 1 light chain 3 beta; GSH, glutathione; Bcl-2, B-cell lymphoma 2; Caspases, cysteine-aspartic proteases; ULK, Unc-51-like kinase; VPS34, vacuolar protein sorting 34; ATG, autophagy-related; GSDMD, gasdermin D; NINJ1, nerve injury-induced protein 1; ESCRT, endosomal sorting complex required for transport; GPX4, glutathione peroxidase 4; FSP1, ferroptosis suppressor protein 1; ACSL4, acyl-CoA synthetase long-chain family member 4; SLC7A11, solute carrier family 7 member 11; FDX1, ferredoxin 1; LIAS, lipoic acid synthetase; DLAT, dihydrolipoamide S-acetyltransferase; SLC31A1, solute carrier family 31 member 1; ATP7A, ATPase copper transporting alpha; ATP7B, ATPase copper transporting beta; PAMPs, pathogen-associated molecular patterns; DAMPs, damage-associated molecular patterns; LPS, lipopolysaccharide; TNF, tumor necrosis factor; PUFA, polyunsaturated fatty acid.

## 4 Mechanism of paraptosis

Paraptosis proceeds via a cascade of events triggered by multiple inducers, ultimately leading to cytoplasmic vacuolization and cell death (**Figure 2**). The basic process of paraptosis involves inducers, such as certain natural compounds (e.g., curcumin, paclitaxel, honokiol, and red ginseng compounds), chemical drugs, nanomedicines, and photodynamic therapy, which induce ER stress (32). This stress leads to swelling of both the ER and mitochondria, which in turn causes changes in the cytoskeleton. These alterations trigger molecular-level changes, including Ca^2+^ imbalance, as well as upregulation of ROS, C/EBP homologous protein (CHOP), Binding immunoglobulin protein (BIP), microtubule-associated protein 1 light chain 3 beta (LC3B), p62, p38, MAPK/p-JNK/p-ERK, and downregulation of Alix, MOMP, adenosine triphosphate (ATP), and glutathione (GSH) (2, 4, 33). These processes ultimately result in the formation of cytoplasmic vacuoles and, eventually, cell death, completing the process of paraptosis.

**Figure 2:**
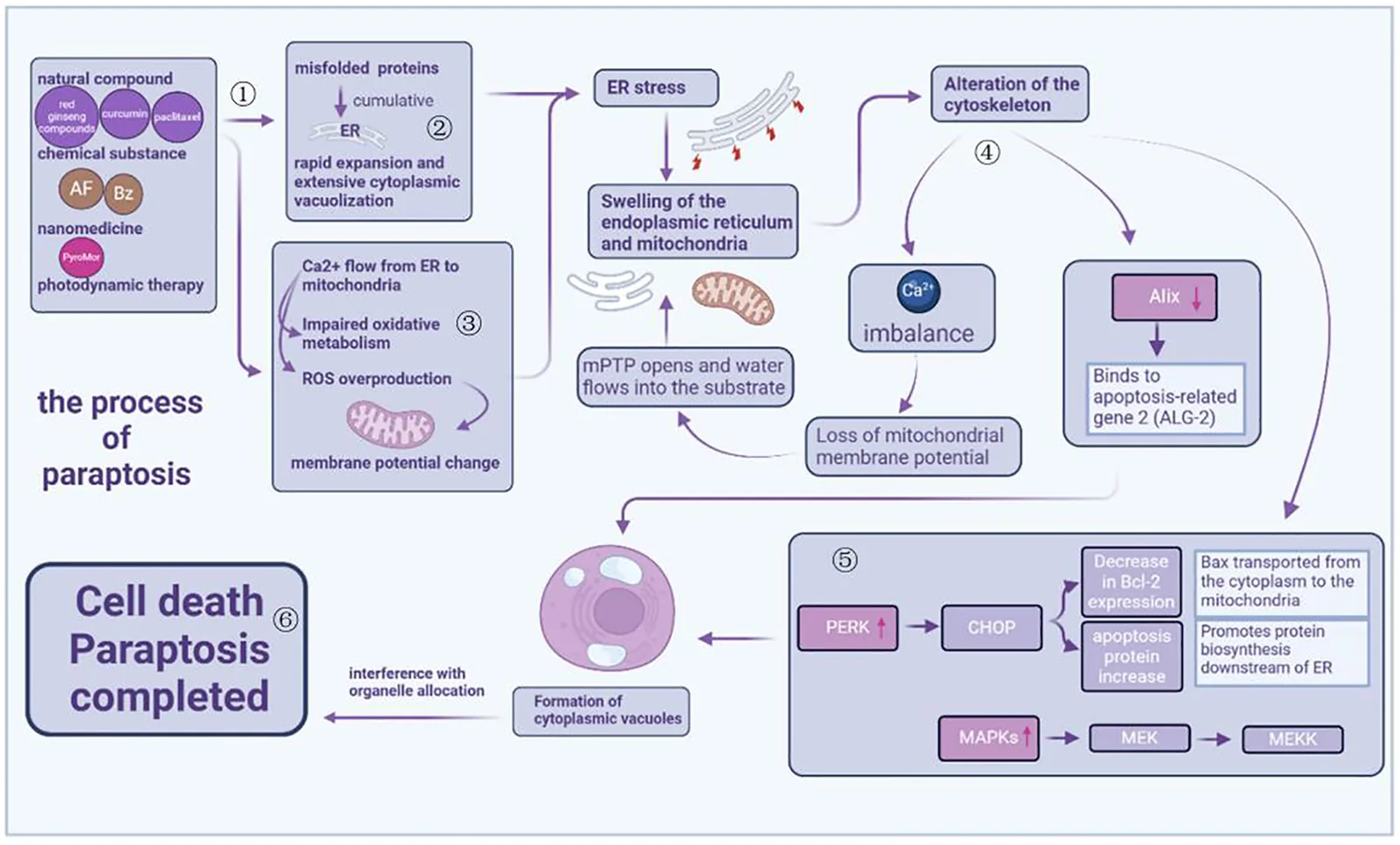
Schematic illustration of the molecular signaling cascade underlying natural product/agent-triggered paraptosis. ① Diverse exogenous stimuli including natural compounds, chemical substances, nanomedicines and photodynamic therapy serve as initiators to trigger paraptosis.② Stimulation drives continuous accumulation of misfolded proteins within endoplasmic reticulum (ER), leading to progressive ER expansion, cytoplasmic vacuolization and persistent ER stress.③Abnormal Ca^2+^ transport from ER into mitochondria impairs mitochondrial oxidative metabolism, induces excessive ROSoverproduction and mitochondrial membrane potential collapse; subsequent swelling of ER and mitochondria opens mitochondrial permeability transition pore (mPTP) and aggravates organelle edema.④ ER-mitochondrial crosstalk causes intracellular Ca^2+^imbalance and cytoskeleton remodeling; downregulated Alix binds to apoptosis-related gene ALG-2 to modulate downstream cell death signals.⑤ Two core downstream signaling cascades are activated upon ER stress: the PERK-CHOP pathway suppresses Bcl-2 expression, promotes Bax cytoplasm-to-mitochondria translocation and accelerates biosynthesis of pro-apoptotic proteins;meanwhile, sequential activation of MAPK-MEK-MEKK cascade amplifies pro-death signaling.⑥ Sustained organelle injury and activated pro-death pathways facilitate massive cytoplasmic vacuole formation, disrupt normal organelle allocation, and ultimately result in the completion of paraptotic cell death.

### 4.1 Abnormal accumulation of misfolded proteins (especially ubiquitinated misfolded proteins)

There is evidence that paraptosis is triggered by misfolded ER proteins and is associated with increased MAPK expression (1, 34–36). Accumulation of misfolded thiol-containing proteins may also contribute to apoptosis induced by dual inhibition of TrxR1 and the proteasome (37, 38). The specific mechanism involves the inhibition of retrotranslocation preventing the protein from entering the cytoplasm through the ER membrane. This disrupts the control and transport system responsible for handling damaged or misfolded proteins. As a result, the misfolded proteins accumulate in the ER, causing vacuole formation and severe ER stress, which leads to ER swelling and ultimately initiates paraptosis (39).

### 4.2 Severe ER stress

Paraptosis is characterized by extensive cytoplasmic vacuolization and swelling of the ER (1). ER stress can be induced by various factors, including accumulation of misfolded proteins in the ER lumen, nutritional deprivation, hypoxia, therapeutic drugs, and inflammatory signals (40). A significant increase in mitochondrial Ca^2+^ levels also contributes to ER stress (5). Following ER stress, the accumulation of misfolded proteins causes a dramatic expansion of the ER and extensive cytoplasmic vacuolization. Large vacuoles disrupt the distribution of organelles, ultimately leading to cell death (41).

### 4.3 Generation of ROS

Paraptosis can be triggered by the generation of reactive oxygen species (ROS) (31, 42). High levels of mitochondrial ROS can induce paraptosis. In δ-tocotrienol-induced paraptosis, the mitochondrial-associated ER membrane facilitates the transfer of Ca^2+^ from the ER to the mitochondria, leading to oxidative metabolic disturbances and excessive ROS production (43). The abnormal increase in ROS alters the mitochondrial membrane potential (44), which is commonly observed in cases of Ca^2+^ overload during pre-paraptosis.

### 4.4 MAPK pathway activation

The MAPK pathway involves three levels of signal transduction: MAPK, MEK, and MEKK (45, 46). These three kinases are sequentially activated and work together to regulate key physiological and pathological processes, including cell growth, differentiation, stress responses, and inflammation (47). The MAPK pathway comprises four main branches: extracellular signal-regulated kinase (ERK), JNK, p38/MAPK, and ERK5.Among these, JNK and p38 share similar functions and are primarily involved in inflammation, apoptosis, and cell growth (48). ERK, on the other hand, is mainly responsible for cell growth and differentiation (49). ERK1/2 can also mediate cell cycle arrest and cell death (50), with its upstream signal originating from the well-known Ras/Raf protein.

In most cases, paraptosis is mediated by MAPKs, such as MEKs or JNKs (51). MAPK signaling plays a crucial role in paraptosis. For instance, knockdown of SHP2 or inhibition of the SHP2/SOS1/MAPK pathway by drug inhibitors significantly attenuates the paraptosis and autophagy inhibition induced by theaflavins (51). Triptolide can also initiate paraptosis by modulating the MAPK signaling pathway (15). Additionally, DET induces apoptosis through both ER stress and MAPK pathways, with the JNK and ERK pathways playing significant roles in this cell death process (52).

### 4.5 Alix

Alix was initially identified as a protein that interacts with the calcium-binding protein ALG-2 (53). It functions as a natural inhibitor of paraptosis and serves as a marker for this process, with the ability to attenuate its initiation (54). The regulation of paraptosis is negatively correlated with the activity of Alix, the ALG-2 interacting protein x (55). For instance, the mulberry-alder adduct Kuwanon M induces paraptosis when Alix is downregulated (56).

Alix may also play a role in apoptosis regulation by binding to the apoptosis-related gene 2 (ALG-2), a Ca^2+^ binding protein essential for cell death. Overexpression of Alix-CT, the carboxyl-terminal domain, can block cell death induced by various stimuli (51). Additionally, overexpression of the Alix-CT fragment inhibits apoptosis under serum-free conditions (57), which might be crucial for Alix-CT’s inhibition of paraptosis. However, Alix-CT overexpression can also cause some ER swelling, impairs the transport of certain ER proteins, and lead to cytoplasmic vacuolization, forming tubular-vesicular structures (53), which may trigger paraptosis. In studies of the anticancer mechanisms of Chalcomoracin, reduced Alix expression was found to coincide with cytoplasmic vacuolization (19).

Therefore, Alix may represent a key point of cross-regulation between apoptosis and paraptosis. Future research should focus on understanding the regulatory mechanisms of Alix, which may offer new strategies for cancer treatment, positioning it as a potential therapeutic target.

### 4.6 Calcium homeostasis

Under normal physiological conditions, the concentration of cytoplasmic Ca^2+^ and Ca^2+^ stored in the ER is tightly regulated. Mitochondria play a crucial role in regulating intracellular Ca^2+^ by sequestering and releasing Ca^2+^ (58, 59).

In paraptosis, disruptions in ion homeostasis appear to play a significant role. Ca^2+^-mediated communication between the ER and mitochondria is critical for apoptosis (60). Mitochondrial Ca^2+^ overload, driven by the intracellular Ca^2+^ flux system along the ER-mitochondrial axis, induces mitochondrial swelling during apoptosis (61–63). Furthermore, the accumulation of misfolded proteins in the ER lumen generates osmotic pressure, drawing water into the ER, which leads to its swelling. During this process, Ca^2+^ released from the ER exacerbates ER stress and contributes to ER expansion (30).

Ca^2+^ and K^+^ are also key players in both physiological and pathological mitochondrial swelling. The imbalance of these ions between the cytosol and mitochondrial matrix increases osmotic pressure, causing water influx and matrix expansion (64). Ca^2+^ leaves the ER via inositol trisphosphate receptors (IP3R) and enters mitochondria through the mitochondrial calcium uniporter (MCU) (65, 66). During paraptosis, the expression of these two receptors increases, enhancing calcium ion transport. The simultaneous expansion of the ER and mitochondria suggests an interconnected relationship between these organelles. The accumulation of mitochondrial Ca^2+^ overload leads to the loss of mitochondrial membrane potential, triggering reactive oxygen species (ROS) production and ultimately, cell death (17, 20, 30, 43).

Long-term mitochondrial Ca^2+^ overload initiates a cascade of events, including the loss of mitochondrial membrane potential, opening of the mitochondrial permeability transition pore (mPTP), and influx of water into the matrix, resulting in mitochondrial swelling (66, 67). Mitochondria cease to absorb Ca^2+^ due to the loss of the electrochemical gradient across the mitochondrial membrane, while the ER also fails to sequester Ca^2+^ because sarcoplasmic/ER Ca^2+^ ATPase is inhibited by ROS and ATP depletion, further elevating cytoplasmic Ca^2+^ levels. Additionally, the excessive depletion of Ca^2+^ from the ER impedes the function of chaperone proteins, which rely on Ca^2+^ binding for protein folding within the ER (68, 69). This leads to an accumulation of misfolded proteins and further osmotic expansion of the ER lumen. This Ca^2+^-mediated vicious cycle between the ER and mitochondria promotes the expansion of mitochondria and ER-derived vacuoles, ultimately resulting in irreversible structural and functional defects in both organelles, culminating in cell death (30).

### 4.7 p-ERK pathway

The activation of the p-ERK signaling pathway occurs early in ER stress and protects cells by promoting cell survival through the inhibition of protein synthesis. As ER stress persists, p-ERK shifts its role to promoting apoptosis by inducing the expression of CHOP (47). Under normal conditions, CHOP is expressed at low levels in the cytoplasm. However, during ER stress, dissociation of BiP/GRP78 and ER membrane proteins such as p-ERK and ATF6 can activate CHOP. CHOP then induces the expression of apoptotic proteins, including GADD34, ERO1, and DR5, which directly activate GADD34 and promote the biosynthesis of ER downstream proteins. Overexpression of CHOP can lead to the generation of reactive oxygen species (ROS), further exacerbating ER stress and mediating apoptosis. Simultaneously, high CHOP expression can reduce Bcl-2 levels, triggering the translocation of Bax from the cytoplasm to mitochondria and initiating the mitochondrial apoptosis pathway (70). Dephosphorylation by p-ERK-specific phosphatases inhibits p-ERK, and the regulation of these signaling pathways plays a crucial role in the pathological processes of the central nervous system, ischemia-reperfusion injury, and metabolic disorders (71).

Hypoxia, a key feature of the tumor microenvironment, impairs the formation of disulfide bonds and disrupts protein folding in the ER. ER stress induced under these conditions activates the unfolded protein response (UPR) pathway via protein kinase RNA-like ER kinase (p-ERK). This leads to a significant increase in phosphorylated eukaryotic initiation factor 2α (p-eIF2α), which promotes the adaptive response by inhibiting global protein synthesis and selectively activating the translation of transcription factor 4 (ATF4). However, prolonged ER stress overwhelms the pro-survival response, resulting in cell apoptosis (72).

### 4.8 Molecular checkpoints governing the choice between paraptosis and apoptosis under therapeutic stress

The mechanism that regulates whether cells undergo apoptosis or paraptosis has not been clearly and completely understood. However, some research mentioned some of the key molecules and signaling pathway. Overproduction of ROS is the initiating event of apoptosis and paraptosis (73, 74). Different targets damaged by ROS, balance of anti-apoptotic and pro-apoptotic proteins in Bcl-2 family, strength of MAPK pathway activation and cell types may be associated with whether cells subsequently undergo apoptosis or paraptosis. Specifically, ER stress and Ca^2+^ overload, upregulated expression of anti-apoptotic proteins in Bcl-2 family, and high-level activation of MAPK signaling pathway may lead to paraptosis. The mechanism may include that ER stress and Ca^2+^ are more likely to activate MAPK pathway, that anti-apoptotic proteins in Bcl-2 family block the release of cytochrome c, and that MAPK activation suppresses caspases activation (2). Furthermore, Protein kinase R-like endoplasmic reticulum kinase (PERK) is considered to play a critical role in the transition from ER stress to paraptosis (40, 75). PERK upregulates the expression of CHOP by activating the PERK-eIF2α-ATF4-SEC61β signaling pathway. Hu et al (75). has reported that drug targeted to PERK of eIF2α can reduce osimertinib-induced CHOP expression; Mandula et al (40). demonstrated that knockdown of PERK may lead to paraptosis. Upregulation of CHOP expression is key signal to suppress autophagy (76). When apoptotic pathway is inhibited, CHOP will be the molecular switch of paraptosis (76). At a further downstream effector level, ERK-2 (a member of the MAPK family) can phosphorylate caspase-9 at Thr125, thereby switching the cell death pathway from apoptosis to paraptosis (2).

## 5 Paraptosis and cancer therapy

Currently, cancer treatment mainly involves surgery, radiotherapy, and chemotherapy, alongside emerging therapies. However, challenges such as drug resistance and the limited availability of targeted drugs continue to hinder effective treatment outcomes. As such, there is a persistent need for new therapeutic strategies. Paraptosis offers a novel approach to inducing cell death, presenting fresh possibilities for cancer treatment and the development of alternative therapies (4). A wide range of drugs, including natural compounds, chemical agents, targeted therapies, and nano-drugs, can induce paraptosis. Moreover, combination therapies using these drugs may enhance paraptosis induction (77). Studies suggest that tumor cells undergoing paraptosis may also trigger an immune response against tumors (78). The prolonged activation of BK channels during paraptosis to kill tumor cells may harness the natural immunogenicity of the tumor cells (40). These findings reveal how p-ERK derived from tumor cells promotes immune evasion and underscore the potential of targeting p-ERK in cancer immunotherapy. Thus, compared to other forms of programmed cell death (PCD), paraptosis may have a more significant impact in cancer immunotherapy. Paraptosis can also be induced by misfolded ER proteins and is associated with increased MAPK expression. Light-induced ER damage has been shown to cause extensive cytoplasmic vacuolation, triggering paraptosis. The use of photodynamic therapy (PDT) to explore apoptosis offers another potential example of employing selective light damage to study death pathways (79).

### 5.1 Drugs that cause paraptosis

A wide range of drugs are capable of inducing paraptosis in the development and progression of various cancers, a phenomenon that provides novel insights and directions for targeted cancer therapy. It is worth noting that the molecular mechanisms by which different drugs trigger paraptosis vary significantly and exhibit certain cancer-type specificity (**Table 2**).

**Table 2:** Drugs that cause paraptosis and the effects.

Category	Agent	Target cancer	Mechanism	Reference
Natural compound	Curcumin, Dimethoxycurcumin (DMC)	Breast cancer, cervical cancer, glioblastoma, gastric cancer	1. Promote vacuolization 2. Regulate Rb, p53, MAPK, PI3K/Akt, JAK/STAT, Shh, and NF-κB signaling pathways	(8, 35, 36, 56, 68)
	Tripterine	Breast cancer, colon cancer, cervical cancer, lung cancer, prostate cancer, non-small cell lung cancer	1. Inhibit proteasome 2. Increase mitochondrial Ca^2+^ levels 3. Induce ER stress 4. Affect the HSP90 pathway	(4, 9, 69)
	Lycopene	Breast cancer	Induce ROS production	(70)
	Bacillus subtilis lipopeptides	Breast cancer, alveolar adenocarcinoma, renal cell carcinoma, colon adenocarcinoma.	Trigger severe ER stress and mitochondrial dysfunction	(3, 71)
	Plumbagin	Breast cancer, cervical cancer, non-small lung cancer	1. Inhibit 26S proteasome chymotrypsin-like activity 2. Induce ER stress	(72)
	6-shogaol	Breast cancer and non-small cell lung cancer	1.Inhibit 26S proteasome chymotrypsin-like activity 2.Induce ER stress	(77, 80)
	Paclitaxel	Ovarian cancer, breast cancer, non-small cell lung cancer	Induce ER stress and mitochondrial Ca^2+^ overload (Together with Honokiol)	(57, 59)
	Honokiol	Lung cancer, leukemia	1.Inhibit proteasome activity 2.Induce ER stress and mitochondrial Ca^2+^ overload	(57, 60, 61, 78, 81)
	Ginsenoside Rh2	Lung cancer, colorectal cancer	Related to p53 activity	(64, 79)
	BEAP	Lung cancer	Induce apoptosis and cell cycle arrest *in vitro* and *in vivo*	(82)
	8-p-Hydroxybenzoyl coumarin	Cervical cancer	Induce ER stress	(83, 84)
	Apigenin	Cervical cancer	1. Induce ER stress 2. Induce crosstalk between apoptosis and autophagy	(68)
	Jolkinolide b	Bladder cancer	Affect thioredoxin and GSH	(85)
	Sanguinarine	Epithelial ovarian cancer	Induce ER stress	(58, 86)
Chemical drugs	Chalcone, 2’-hydroxy-chalcone(HRC)	Lung cancer, breast cancer, cervical cancer	1. Induce ER stress, 2. Inhibit proteasome chymotrypsin-like activity 3. Cause mitochondrial membrane potential abnormality, 4. Lead to ATP depletion	(65, 87)
	δ-TT	Prostate cancer	Impair mitochondrial function	(88, 89)
	Auranofin	Breast cancer	1. Inhibit thioredoxin reductase 1 (TrxR1) 2. Inhibit proteasome	(26)
	Indirubin-3’-monoxime	Breast cancer	Induce ER stress	(90)
	Bortezomib	Breast cancer	Inhibit proteasome	(91)
	HQ-11	Breast cancer	Cause ER and mitochondrial vacuolization	(92)
	Active platinum complex	Lung cancer, rectal cancer	1. Disrupt redox balance 2. Induce ER stress and mitochondrial dysfunction	(93)
Targeted drugs	ReLMito	-	Affect mitochondria	(94)
	DETD-35	Triple-negative breast cancer	1. Induce increased ROS production 2. Disrupt ER structure	(41)
	Hep88 mAb-mortalin compound	Hepatocellular carcinoma	Bind to Mortalin	(95)
	4-PQBH	Hepatocellular carcinoma	Regulate Nur77-dependent cytoplasmic vacuolation and paraptosis	(96)

DMC dimethoxycurcumin, ER endoplasmic reticulum, HRC 2’-hydroxy-chalcone.

#### 5.1.1 Proteasome inhibitor

Proteasome inhibitors are substances that bind to specific groups on the active site of protease molecules, leading to a reduction or complete loss of protease activity. They have already been used in the treatment of hematological malignancies, for instance, bortezomib is the first-line therapeutic option of multiple myeloma(MM) and mantle cell lymphoma, and carfilzomib is mainly used in relapsed or refractory MM (97, 98). Previous studies have shown that proteasome inhibitors exhibit cytotoxicity via promoting intrinsic and extrinsic apoptosis. Strauss et al (99). showed that, in the sensitive cell lines, bortezomib suppresses the NF-κB pathway and upregulates the pro-apoptotic Bcl-2 proteins by inhibiting the chymotryptic activity. This leads to the change in mitochondrial membrane potential, triggering the release of cytochrome c and caspase activation, and eventually culminates in apoptosis. Furthermore, in the most sensitive cell line (DHL-7), researchers find that bortezomib downregulates Cyclin B and Wee-1, resulting in mitotic catastrophe. Carfilzomib is representative of the second generation proteasome inhibitor. Jarauta et al (100). demonstrated the mechanism of using carfilzomib to treat MM. Carfilzomib can upregulate Puma and Noxa proteins, promoting the interaction between Puma, Noxa and Bim, Bak, as well as interaction between Noxa and Bak, and eventually leads to apoptosis. Other studies have shown that the mechanism that proteasome inhibitors lead to apoptosis may be related to ER stress (101). They impair the clearance of misfolded proteins, therefore induce the accumulation of misfolded protein, which may activate the C/EBP homologous protein (CHOP), upregulating the expression of Bim and suppressing the Bim degradation, therefore activates the apoptotic signaling pathway (101).

However, existing studies on paraptosis have also indicated that inhibition of proteasomes is a critical factor in triggering paraptosis (37). The disruption of protease activity and the resultant Ca^2+^ imbalance induced by proteasome inhibitors can promote paraptosis in cells. By disrupting protein homeostasis, protease inhibitors induce stress in both the ER and mitochondria (20), leading to cytotoxic effects in cancer cells (37) and ultimately resulting in paraptosis. However, tumor cells can also prevent paraptosis by activating other protective signaling pathways. Recent findings by Zhang et al. demonstrate that proteasome inhibition activates the protein kinase CaMKII, which enhances the integrated stress response (ISR) by phosphorylating BAG3 in tumor cells and reduces the production of misfolded proteins, thereby inhibiting paraptosis (82). This suggests that targeting the ISR may represent a potential strategy to sensitize tumor cells to proteasome inhibitor-induced paraptosis.

#### 5.1.2 Curcumin

Curcumin interacts with several oncogenic and tumor-suppressing miRNAs involved in various stages of breast cancer (83). It regulates pathways such as p53, MAPK, JAK/STAT, and NF-κB (85), and selectively induces mitochondrial Ca^2+^ overload by promoting vacuolization, which results from mitochondrial and/or ER expansion and fusion (14, 87). This process ultimately triggers paraptosis, contributing to its anti-tumor effects. Curcumin is considered a pharmacologically safe compound that can be used for the chemoprevention and treatment of cancer (14). The use of curcumin to induce paraptosis offers a promising, safe anticancer strategy.

Current research showed that curcumin oral formulations and liposomal intravenous administration have shown favorable safety profiles. Kim et al (102). showed that liposomal curcumin has a good safety profile. Pharmacokinetically, oral curcumin formulations suffer from low bioavailability, which nano-liposomal preparations can significantly improve (102). The combination of curcumin with radiotherapy and temozolomide for the treatment of newly diagnosed high-grade gliomas has entered the phase I/II clinical trial stage (103).

#### 5.1.3 Paclitaxel

The combination of paclitaxel and honokiol can synergistically induce tumor cell death by triggering paraptosis. Treatment with paclitaxel and honokiol significantly delays the growth of H1299 xenograft tumors and induces extensive cytoplasmic vacuolation (88). Disruption of intracellular Ca^2+^ balance and mitochondrial Ca^2+^ overload, leading to mitochondrial dysfunction, has also been implicated in paclitaxel/honokiol-induced paraptosis (90). The effects of different concentrations of paclitaxel on the form of cell death vary. While the mechanism behind high-concentration paclitaxel-induced paraptosis remains unclear, this finding holds potential significance for cancer treatment, particularly for overcoming resistance in apoptotic drug-resistant cancers (104).

The primary dose-limiting toxicity of paclitaxel is neutropenia, and it has a relatively favorable safety profile (105, 106). It has already been widely used in the treatment of solid tumors such as breast cancer, non-small cell lung cancer, and ovarian cancer.

#### 5.1.4 Honokiol

Honokiol (HNK) induces a novel form of cell death in human leukemia NB4 and K562 cells (92). This alternative cell death, activated by low concentrations of honokiol, meets the criteria for paraptosis. Notably, honokiol-induced paraptosis does not involve membrane lysis, chromatin condensation, or phosphatidylserine exposure on the outer plasma membrane. Further analysis reveals that in NB4 cells, honokiol primarily induces cell death through paraptosis. These findings suggest that paraptosis could serve as a promising therapeutic pathway for treating leukemia with honokiol (107).

Current trials have shown that HNK has poor water solubility and poor absorption, and the use of liposomes is a potential strategy to improve its bioavailability (108). A phase I trial evaluating the safety of the dietary supplement honokiol for lung cancer chemoprevention is in the patient recruitment stage, no relative results have been reported.

#### 5.1.5 Red ginseng compound (RAG)

The primary anti-cancer mechanisms of red ginseng compounds include cell cycle arrest, induction of apoptosis/paraptosis, and inhibition of angiogenesis (94). The combination of 5-FU and RAG exhibits a synergistic effect. Cell cycle analysis revealed that 5-FU induces S phase arrest, whereas the combination of 5-FU and RAG increases the proportion of cells in G1 phase. Thus, the enhanced anti-cancer effect of RAG in combination with 5-FU is associated with the induction of both paraptosis and apoptosis (95).

Current clinical trials did not report occurrence of adverse events, and RAG has already been widely used as dietary supplement (109, 110).

#### 5.1.6 Protopanaxanol (PPD)

PPD induces colorectal cancer cell death by triggering paraptosis. The proportion of cytoplasmic vacuoles in cancer cells treated with PPD was significantly increased, and mitochondrial swelling assays further confirmed the paralysosomal induction by PPD. Additionally, PPD induces ROS production in cancer cells, which activates the NF-κB pathway (96, 111).

PPD exhibited low toxicity in preclinical trial (112). However, due to its poor water solubility, its oral bioavailability is limited in pharmacokinetic evaluations (104).

#### 5.1.7 Chalcone

2’-Hydroxy-chalcone (HRC) effectively induces apoptosis in malignant breast and cervical cancer cells. Studies have shown that HRC binds to the Thr1 residue at the active site of the proteasome’s chymotrypsin-like subunit. Fluorescence-based assays targeting the 26S proteasome chymotrypsin-like subunit further confirm HRC’s inhibitory effect on proteasome activity. Additionally, the collapse of mitochondrial membrane potential and ATP depletion contribute to HRC-induced cell death. These findings suggest that HRC can effectively eliminate cancer cells through the alternative death pathway of paraptosis and may serve as a potential lead compound for anticancer therapy (113).

For chalcones, trials are mainly concentrated in the preclinical research stage, and the toxicity remains unclear (114). These compounds commonly suffer from poor bioavailability and rapid clearance (115).

#### 5.1.8 Glabrene

Glabrene has been shown to possess anti-tumor properties in human malignant tumors (116, 117). It reduces the survival rate of human breast cancer MDA-MB-231 and MCF7 cells in a dose-dependent manner. By upregulating the expression of ER stress markers such as BiP, XBP1s, and CHOP, Glabrene promotes the formation of extensive cytoplasmic vacuolization derived from the ER, thereby inducing paraptosis (116). However, glabrene has demonstrated significant toxicity in preclinical studies, which may restrict its potential for clinical application (118).

### 5.2 Combined use of drugs

The combination of drugs in cancer treatment may lead to improved therapeutic outcomes and has become a promising alternative and effective strategy for cancer chemotherapy. For example, the combination of perifosine and vitamin D can synergistically enhance the expression of p53 mRNA, thereby inducing apoptosis in HEC-1A cells and ultimately resulting in both apoptotic and non-apoptotic cell death (including para-apoptotic, autophagic, and necrotic death) (77). The novel combination of CIB1 depletion with docetaxel or TRAIL selectively enhances cell death in both naive and docetaxel-resistant TNBC cells while sparing normal cells (119). Red ginseng enhances the effectiveness of fluorouracil in human colon cancer cells through both paraptosis and apoptosis pathways (95). TACC3 protein induces cytoplasmic vacuolization, sensitizing cancer cells to bortezomib. Paraptosis induced by TACC3 can be utilized to treat apoptosis-resistant cancer cells through XIAP overexpression (120). The combination of docosahexaenoic acid and sodium selenite promotes paraptosis in colorectal cancer cells by disrupting redox homeostasis and activating the MAPK pathway, providing a new strategy for more targeted and less toxic cancer therapy (121). Vitamin B12b causes cytoplasmic vacuolization, mitochondrial dysfunction, and increased superoxide anion production, which synergistically enhances the cytotoxicity of diethyldithiocarbamate and induces paraptosis-like cell death in human laryngeal cancer cells (122).

Additionally, potent paraptosis-inducing nanomedicines are emerging that can trigger rapid non-apoptotic death in cancer cells, overcoming apoptosis-based drug resistance, and effectively inhibiting the growth of drug-resistant tumors. These nanomedicines primarily exert anti-tumor effects by increasing intracellular ROS levels, causing cytoplasmic vacuolization, and promoting organelle swelling (123). For instance, the nanomedicine PyroMor can be effectively internalized by tumor cells and generate ROS, inducing apoptosis under light irradiation. The release of Mor from PyroMor further leads to severe vacuolization of the ER and mitochondria, triggering paraptosis, which can be used for combined tumor therapy (124). Another example is an HQ-based Cu^2+^-responsive polymer that forms micelles and utilizes the EPR effect to accumulate in tumors. In the tumor, Cu^2+^ catalyzes the hydrolysis of the HQ-conjugated linker to form Cu(HQ)_2_, effectively inducing near-apoptotic death in drug-resistant tumor cells (125).

### 5.3 Immunotherapy

Some previous treatments can stimulate autophagy in cancer cells, but certain cancer cells can survive and develop resistance to these treatments. The ability to evade programmed cell death processes, for example, apoptosis, is significant in the development of resistance (126). Ergün et al (127). mentioned the core mechanisms of apoptosis evasion include: Activation of Bcl-2 family anti-apoptotic proteins leads to resistance of anoikis; Mitochondrial DNA depletion may cause decreased ROS production and inhibition of pro-apoptotic factors release; The upregulation of c-FLIP competes with caspase-8 for binding to FAS, thereby suppressing the extrinsic apoptotic pathway; The endosomal sorting complexes required for transport mechanism repair the damaged plasma membrane; Mitotic slippage, anastasis and blebbishield formation have also been implicated in apoptosis evasion. Apoptosis evasion may leads to the absence of ICD, shaping the immunosuppressive microenvironment (126). This mechanism substantially contributes to the limitation of immunotherapy (126). Breast cancer, for example, has been classified as ‘immune-cold’ or ‘immune-inert’ since the 1980s (128). Converting “cold tumors” into “hot tumors” is the core of enhancing the efficacy of immunotherapy (129). Previous studies have demonstrated that in various “cold tumors”, paraptosis can enhance immune activation by inducing immunogenic cell death (ICD), and plays a significant role in overcoming immunosuppression and drug resistance (10, 40).

Numerous studies have shown that prolonged activation of apoptosis can enhance the immunogenicity of tumors (6). This increased immunogenicity can trigger the activation of cellular immunity against cancer cells, leading to their destruction. Antigen processing and presentation enables the immune system to monitor cellular processes and respond when abnormal or foreign proteins are expressed. Paraptosis can also mediate immunogenic cell death (ICD) in cancer cells. ICD refers to cancer cell death that locally and systemically triggers anti-tumor immune responses. This process is characterized by the release of intracellular ATP, high mobility group protein 1 (HMGB1), the externalization of calreticulin to the cell membrane (ExoCRT), and the production of type I interferon (IFN) (130). Paraptosis can lead to ER stress, subsequently releasing ICD signals through p-ERK activation (131). The intensity of ER stress positively correlates with the immunogenicity of the resulting cell death (132). For example, in para-apoptotic tumor cells, the elimination of p-ERK triggers Sec61β-induced apoptosis, thus promoting ICD and systemic anti-tumor responses (40). Additionally, paraptosis is often accompanied by an increase in ROS, which has been found to induce ICD through ER stress. For instance, toluidine can drive ROS-mediated ER stress-induced ICD in hepatocellular carcinoma (133). Since paraptosis does not rely on the classical apoptotic molecules like caspases and Bcl-2 family proteins, it can avoid most of apoptosis evasion mechanism (31, 134). Promoting paraptosis, in this sense, holds significant potential in modulating the immune microenvironment, facilitating immune cell recruitment and activation, and thus improving immunotherapeutic outcomes.

Thus, promoting paraptosis can enhance immunogenic cell death, a form of tumor cell death that can be harnessed in immunotherapy.

### 5.4 Photodynamic therapy

Photodynamic therapy (PDT) is a photocatalytic process that traditionally induces cell death through three main pathways: apoptosis, autophagy-related cell death, and necrosis. PDT generates cytotoxic reactive oxygen species (ROS) at specific subcellular sites, which then initiate the cell death process (135). Recent studies have revealed that cancer cells exposed to PDT can undergo various unconventional cell death pathways, such as paraptosis, mitotic catastrophe, thermal apoptosis, necrosis, and iron-induced apoptosis (136). Light damage to the ER and liposomes has been shown to induce apoptosis, and PDT has been utilized to treat both cancer and non-malignant diseases (136, 137). For example, using hypericin as a photosensitizer, researchers have observed the formation of vacuoles around the nucleus as early as 4 hours after the LD90 PDT dose, suggesting that paraptosis occurred (79).

Studies indicate that mild ER light damage leads to the activation of the classical apoptotic pathway. However, when the ER damage approaches the LD90 level, the cell death pathway deviates from the classical model: interfering with protein synthesis does not prevent cell apoptosis, and transient low temperatures following irradiation fail to protect cells. MAPK signaling is not involved, and stimulating autophagy does not provide cytoprotective effects (138). PDT employs a photosensitizer (PS) that is activated by light at a specific wavelength in the presence of oxygen. The excited PS binds to target cells and induces oxygen production, resulting in local light damage and subsequent cell death (139). The photodynamic process can be divided into two stages: (1) PSs absorb light and are excited to a higher energy state; (2) The excited PSs relax and transfer energy to molecular oxygen, generating ROS, singlet oxygen (^1^O_2_), and other free radicals. These molecules cause damage to DNA, membranes, and other organelles in cells in two ways. In the first scenario, ^3^PS* transfers electrons and protons from nearby molecules (e.g., polyunsaturated fatty acids in cell membranes). These electrons and protons then interact with cellular oxygen to produce cytotoxic ROS, such as superoxide anions (O^2-^), hydrogen peroxide (H_2_O_2_), and hydroxyl radicals (OH^-^), triggering free radical chain reactions (140). The second reaction type involves the transfer of energy from 3PS to molecular oxygen, resulting in the formation of ^1^O_2_, a potent oxidant. Both types of reactions can occur simultaneously, with their ratio depending on the photochemical and photophysical properties of the PS and cellular oxygen. Ultimately, these reactions activate various cell death mechanisms in cancer cells through different pathways, leading to tumor tissue inhibition (141, 142).

## 6 Clinical translation of paraptosis-inducing agents

### 6.1 Conclusions and outlook

Paraptosis is a non-cysteinyl aspartic acid-specific protease-dependent cell death pathway characterized by vacuolization and damage to the ER and mitochondria. It is typically induced by factors such as the expression of insulin-like growth factor 1 receptor (IGF1R), proteasome inhibition, ER stress, reactive oxygen species (ROS) production, and Ca^2+^ overload. These factors lead to extensive cytoplasmic vacuolization and the expansion of the ER and mitochondria, ultimately resulting in paraptosis.

However, there are still many challenges in the research on paraptosis. To begin with, the molecular mechanism of paraptosis has not been explicitly demonstrated. Particularly, the molecular switch that determines whether cells undergo paraptosis versus apoptosis remains to be elucidated and specific apoptotic biomarkers remain unclear. Furthermore, there is no well-established, safe, and effective strategy for overcoming tumor drug resistance via paraptosis. Even though many *in vitro* experiment demonstrated that drug can induce apoptosis and kill the drug-resistant tumor cells, two major challenges remain to be overcome: Whether that strategy is effective *in vivo* and how we make sure the drug has no off-target toxicity. Moreover, many research have reported different types of tumor cells has different sensitivity, which may leads to limitation of the application of this strategy. Ultimately, limited number of related studies leads to a weak foundation for new research. The future research may concentrate on the mechanism, especially specific biomarkers and molecular switch, *in vivo* experiment and translational research step by step to develop this promising new therapeutic strategy in the field of tumor therapy.

In cancer treatment, paraptosis holds potential for immunotherapy, as it can inhibit the immune escape of cancer cells. It may also play a role in radiotherapy and chemotherapy. Currently, paraptosis is primarily induced in immunotherapy and PDT for cancer treatment. However, there is still insufficient research in this area, and the types of drugs and cancers suitable for inducing paraptosis remain limited. The specific therapeutic effects, as well as the potential toxicities and side effects of therapies that induce paraptosis, are not well understood. The mechanisms of targeted therapy for paraptosis are also unclear. More research is needed to address these issues.

In conclusion, as a novel form of cell death and a potential new target for tumor therapy, paraptosis shows great promise in cancer treatment and warrants further in-depth investigation.
